# Comparative cytogenetics among *Leporinusfriderici* and *Leporellusvittatus* populations (Characiformes, Anostomidae): focus on repetitive DNA elements

**DOI:** 10.3897/CompCytogen.v13i2.33764

**Published:** 2019-04-05

**Authors:** Thais Aparecida Dulz, Carla Andrea Lorscheider, Viviane Demetrio Nascimento, Rafael Bueno Noleto, Orlando Moreira-Filho, Viviane Nogaroto, Marcelo Ricardo Vicari

**Affiliations:** 1 Programa de Pós-Graduação em Genética, Universidade Federal do Paraná, Centro Politécnico, Jardim das Américas, 81531-990, Curitiba, Paraná State, Brazil Universidade Federal do Paraná Curitiba Brazil; 2 Departamento de Ciências Biológicas, Universidade Estadual do Paraná, Praça Coronel Amazonas, s/n°, 84600-185, União da Vitória, Paraná State, Brazil Universidade Estadual do Paraná União da Vitória Brazil; 3 Departamento de Genética e Evolução, Universidade Federal de São Carlos, Rodovia Washington Luís, Km 235, 13565-905, São Carlos, São Paulo State, Brazil Universidade Federal de São Carlos São Carlos Brazil; 4 Departamento de Biologia Estrutural, Molecular e Genética, Universidade Estadual de Ponta Grossa, Av. Carlos Cavalcanti, 4748, 84030-900, Ponta Grossa, Paraná State, Brazil Universidade Estadual de Ponta Grossa Ponta Grossa Brazil

**Keywords:** Chromosomal differentiation, karyotype evolution, ribosomal DNA, retrotransposon

## Abstract

Anostomidae are a neotropical fish family rich in number of species. Cytogenetically, they show a conserved karyotype with 2n = 54 chromosomes, although they present intraspecific/interspecific variations in the number and chromosomal location of repetitive DNA sequences. The aim of the present study was to perform a comparative description of the karyotypes of two populations of *Leporinusfriderici* Bloch, 1794 and three populations of *Leporellusvittatus* Valenciennes, 1850. We used conventional cytogenetic techniques allied to fluorescence *in situ* hybridization, using 18S ribosomal DNA (rDNA) and 5S rDNA, a general telomere sequence for vertebrates (TTAGGG)n and retrotransposon (RTE) *Rex1* probes. The anostomids in all studied populations presented 2n = 54 chromosomes, with a chromosome formula of 32m + 22sm for *L.friderici* and 28m + 26sm for *L.vittatus*. Variations in the number and location of the 5S and 18S rDNA chromosomal sites were observed between *L.friderici* and *L.vittatus* populations and species. Accumulation of *Rex1* was observed in the terminal region of most chromosomes in all populations, and telomere sequences were located just on all ends of the 54 chromosomes in all populations. The intraspecific and intergeneric chromosomal changes occurred in karyotype differentiation, indicating that minor chromosomal rearrangements had present in anostomid species diversification.

## Introduction

Eukaryotic chromosomes can be classified into different DNA classes: single copy DNA, which are sequences found only once in a genome; and repetitive DNA, which are sequences repeated from a few tens to millions of times (Sumner 2003). Repetitive DNA can be classified into tandem repeats (multigene families and satellite, minisatellite, and microsatellite DNA) and transposable elements (TEs): transposons and retrotransposons with dispersed distribution in genomes (Sumner 2003).

Satellite DNA and TEs are responsible for a large part of the structural and functional organization of genomes (Sumner 2003, Feschotte 2008), and carry sequences containing DNA double-strand break hotspots, resulting in chromosome/genome reshuffle (Eichler and Sankoff 2003, Longo et al. 2009, Farré et al. 2011, Barros et al. 2017a, Glugoski et al. 2018). The movement of repetitive sequences within the genome promotes chromosomal differentiation, which has an important role on karyotype evolution (Wichman et al. 1991, Pucci et al. 2016, 2018a, 2018b, Lorscheider et al. 2018, do Nascimento et al. 2018).

Anostomids are neotropical fishes with a high number of species and diverse morphology (Garavello and Britski 2003, Graça and Pavanelli 2007, Britski et al. 2012, Ramirez et al. 2017a). Cytogenetically they present a conserved diploid number (2n) of 54 chromosomes, with mostly metacentric (m) and submetacentric (sm) chromosomes (Galetti Jr and Foresti 1986, Galetti Jr et al. 1991, 1995, Venere et al. 2004). Anostomidae species present differentiated karyotypes regarding the distribution of heterochromatin and repetitive sequences, presenting different localizations of heterochromatic bands and repetitive DNA sites (Martins and Galetti Jr 1999, Parise-Maltempi et al. 2007, Porto-Foresti et al. 2008, Hashimoto et al. 2009, Marreta et al. 2012, Borba et al. 2013).

Therefore, although they retain 2n = 54 chromosomes, anostomids present very high intra- and interspecific chromosomal/genetic variability, which is highly compatible with restricted gene flow (Parise-Maltempi et al. 2007, 2013, Ramirez et al. 2017a, 2017b, Silva-Santos et al. 2018). With the aim of better understanding the intra- and interspecific chromosomal differentiation due to accumulation of repetitive sequences, in the present study we performed a comparative evaluation of the karyotypes of two populations of *Leporinusfriderici* (Bloch, 1794) and three populations of *Leporellusvittatus* (Valenciennes, 1850). Cytogenetic analysis was performed using Giemsa staining and C-banding, and chromosome mapping of repetitive DNAs using the ribosomal DNA (rDNA) 18S and 5S rDNA, the (TTAGGG)n sequence and the retrotransposon (RTE) *Rex1*.

## Material and methods

Specimens of *Leporinusfriderici* and *Leporellusvittatus* were collected from rivers belonging to different Brazilian hydrographic basins (Table 1). Fish capture was authorized by the Instituto Chico Mendes de Conservação da Biodiversidade (ICMBio – license numbers 10538-1 and 15117-1) and the processing was performed in accordance with the Ethical Committee on Animal Use (CEUA 29/2016) of the Universidade Estadual de Ponta Grossa and current Brazilian legislation. The analyzed specimens were identified by taxonomists experts in the Núcleo de Pesquisas em Limnologia, Ictiologia e Aquicultura (Nupelia) museum, Universidade Estadual de Maringá (UEM).

**Table 1.** Cytogenetic data of *Leporinusfriderici* and *Leporellusvittatus* analyzed in the present study. SP = São Paulo State, PR = Paraná State, MG = Minas Gerais State, MT = Mato Grosso State, 2n = diploid number, FN = fundamental number, KF = karyotype formula, term = terminal sites.

Species	River/Basin/State/GPS	2n	FN	KF	5S sites	18S sites	*Rex1*
* Leporinus friderici *	Mogi-Guaçu River, Upper Paraná Basin – SP (21°58'52"S, 47°17'36"W)	54	108	32m+22sm	pairs 10 and 11	pair 1	term
	Jangada River, Iguaçu River Basin – PR (26°13'5.22"S, 51°16'17.40"W)	54	108	32m+22sm	pairs 3 and 11	pair 1	term
* Leporellus vittatus *	Mogi-Guaçu River, Upper Paraná Basin – SP (21°58'52"S, 47°17'36"W)	54	108	28m+26sm	pair 3	pair 5	term
	Aripuanã River, Aripuanã River Basin – MT (10°09'57.8"S, 59°26'54.9"W)	54	108	28m+26sm	pairs 6 and 8	pair 6	term
	São Francisco River, São Francisco Basin – MG (20°16'15"S, 45°55'39"W)	54	108	28m+26sm	pair 3	pair 6	term

Genomic DNA was extracted from the liver tissue, using the protocol of Doyle and Doyle (1990), from the *Megaleporinusobtusidens* (Ramirez et al. 2017a), described first time in the literature as *Leporinusobtusidens* (Valenciennes, 1837). The 18S rDNA amplification was performed using primers 18S Fw (5’-ccgctttggtgactcttgat-3’) and 18S Rv (5’-ccgaggacctcactaaacca-3’), according to Gross et al. (2010). The 5S rDNA sequence was amplified using primers 5SA (5’-tcaaccaaccacaaagacattggcac-3’) and 5S (5’-tagacttctgggtggccaaaggaatca-3’), according to Martins and Galetti (1999). The vertebrate telomere sequence (TTAGGG)n was obtained according to Ijdo et al. (1991). The non-long terminal repeats retrotransposon (non-LTR RTE) *Rex1* sequence was obtained by PCR using primers RTX1-F1 Fw (5’-ttctccagtgccttcaacacc-3’) and RTX1-R1 Rv (5’-tccctcagcagaaagagtctgctc-3’), according to Volff et al. (1999, 2000). The sequences of the 5S rDNA, 18S rDNA and *Rex-1* were analyzed and their nucleotide identities were confirmed using BLASTn (National Center for Biotechnology Information) and the CENSOR tool for repeated sequences (Kohany et al. 2006). Finally, the sequences were deposited in GenBank (Sequences ID: MH697559, MH701851, MH684488, respectively).

Mitotic chromosomes were obtained according to Blanco et al. (2012) and stained with 5% Giemsa in phosphate buffer, pH 6.8. Heterochromatin detection was performed according to Sumner (1972), with modifications (Lui et al. 2009).

The 18S rDNA was labeled with digoxigenin-11-dUTP, using the DIG-Nick Translation Mix (Roche Applied Science), according to the manufacturer’s recommendations. The 5S rDNA sequence was labeled with biotin 16-dUTP by PCR, and *Rex1* and (TTAGGG)n sequences with digoxigenin-11-dUTP by PCR. PCR reactions were performed with 20 ng DNA template, 1× polymerase reaction buffer, 1.5 mM MgCl2, 40 µM dATP, dGTP and dCTP, 28 µM dTTP, 12 µM digoxigenin-11- dUTP or biotin 16 dUTP, 1 µM of each primer and 1 U of DNA Taq polymerase. The PCR program consisted of an initial step of denaturation at 95 °C for 5 min, 30 cycles of 95 °C for 30 s, 56 °C for 45 s, 72 °C for 2 min, and a final extension at 72 °C for 7 min.

The general protocol for FISH (Pinkel et al. 1986) followed under hybridization mixture (2.5 ng/μl probe, 50% formamide, 2×SSC, 10% dextran sulfate, at 37 °C for 16 h). Post-hybridization washes were performed in high stringency [50% formamide at 42 °C for 10 min (twice times), 0.1×SSC at 60 °C for 5 min (three times), and 4×SSC 0.05% Tween at room temperature for 5 min (two baths)]. Streptavidin Alexa Fluor 488 (Molecular Probes) and Anti-digoxigenin rhodamine fab fragments (Roche Applied Science) antibodies were used for probes detection. The chromosomes were stained with DAPI (0.2 μg/ml) in Vectashield mounting medium (Vector) and analyzed under epifluorescence microscopy.

Chromosome preparations were analyzed using the brightfield and epifluorescence microscope Zeiss Axio Lab 1, coupled to the Zeiss AxioCam ICM1 camera with the Zen Lite software and a resolution of 1.4 megapixels (Carl Zeiss). The karyotypes were organized and classified as metacentric (m) or submetacentric (sm) according to Levan et al. (1964).

## Results

**Figure 1.**
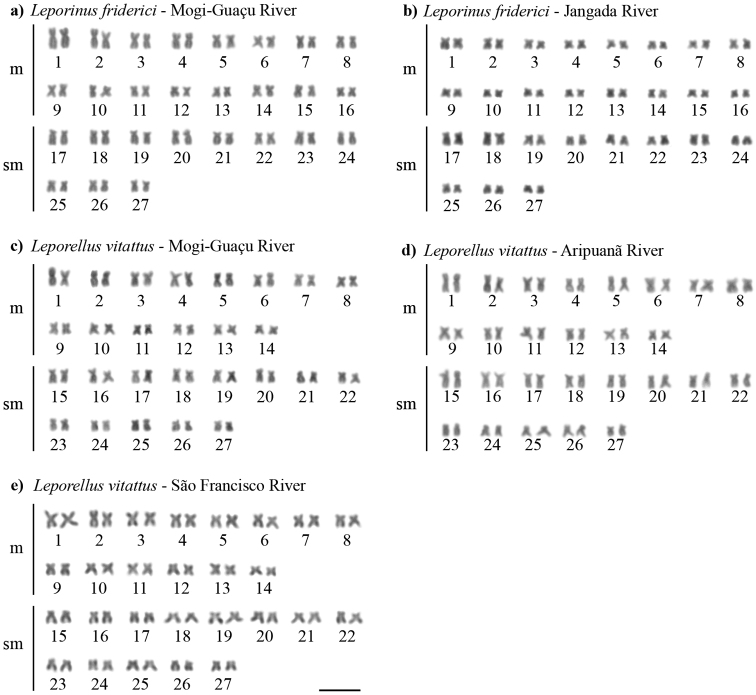
Karyotypes of *Leporinusfriderici* (**a, b**) and *Leporellusvittatus* (**c, d, e**) after conventional Giemsa staining. Scale bar: 10 µm.

All anostomids evaluated in the present study presented 2n = 54 chromosomes and a fundamental number (FN) of 108 (Table 1). The two populations of *L.friderici* (Mogi–Guaçu and Jangada rivers) presented a karyotype formula (KF) of 32m + 22sm (Fig. 1a, b), and the three populations of *L.vittatus* (Mogi–Guaçu, Aripuanã and São Francisco rivers) a karyotype formula of 28m + 26sm (Fig. 1c, d, e). Sex chromosome heteromorphism was not detected in the populations/species analyzed.

**Figure 2.**
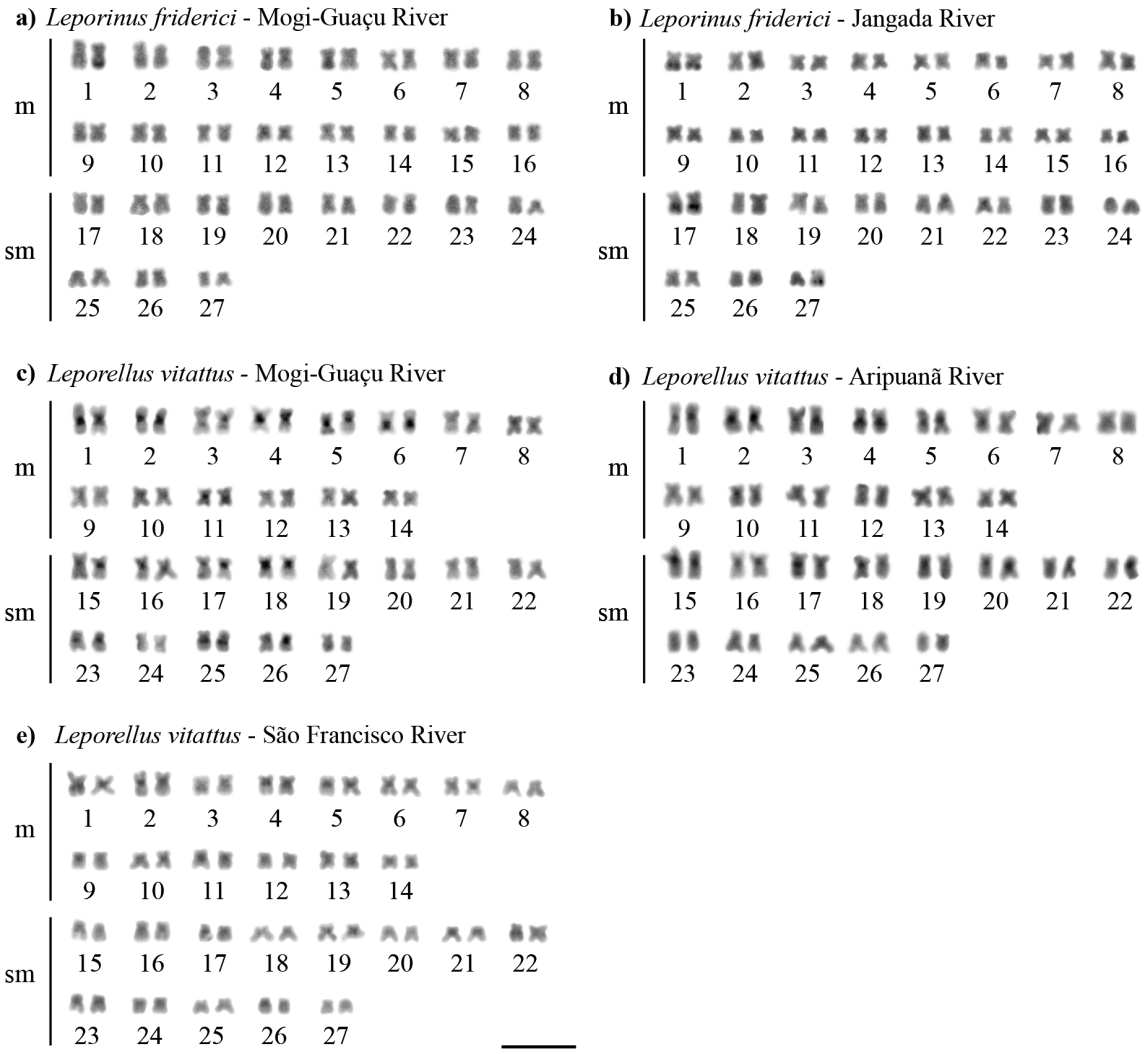
Karyotypes of *Leporinusfriderici* (**a, b**) and *Leporellusvittatus* (**c, d, e**) after C-banding. Scale bar: 10 µm.

C-banding showed discrete blocks of centromeric heterochromatin for *L.friderici*, with very evident blocks in the terminal regions of the long arms of just one homologue of chromosomes 1 and 5 for the population of the Mogi–Guaçu river (Fig. 2a); and, in the subterminal regions of pairs 1 and 17 for the population of the Jangada river (Fig. 2b). *Leporellusvittatus* showed blocks of heterochromatin in the pericentromeric or proximal regions of most chromosomes (Fig. 2c, d), which was very evident for the populations from the Mogi–Guaçu and Aripuanã rivers and less evident for the populations from the São Francisco river (Fig. 2e).

**Figure 3.**
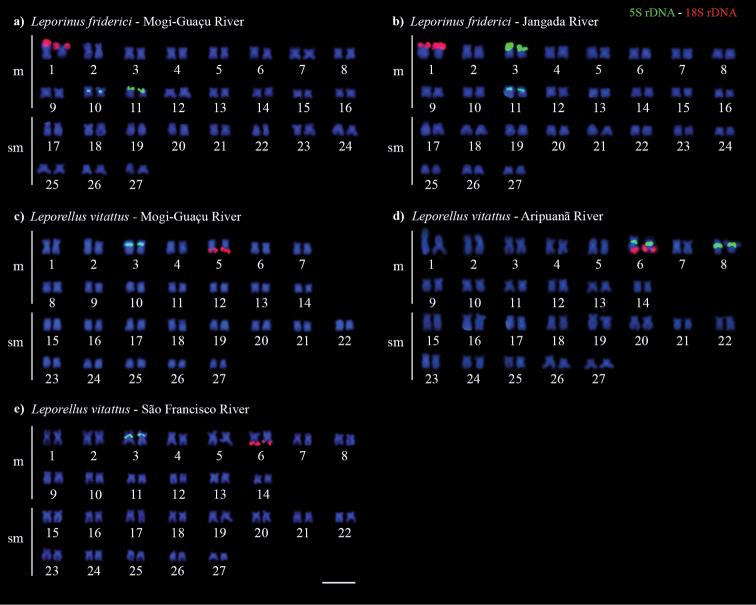
Karyotypes of *Leporinusfriderici* (**a, b**) and *Leporellusvittatus* (**c, d, e**) submitted to fluorescence *in situ* hybridization with 18S rDNA and 5S rDNA probes. Scale bar: 10 µm.

**Figure 4.**
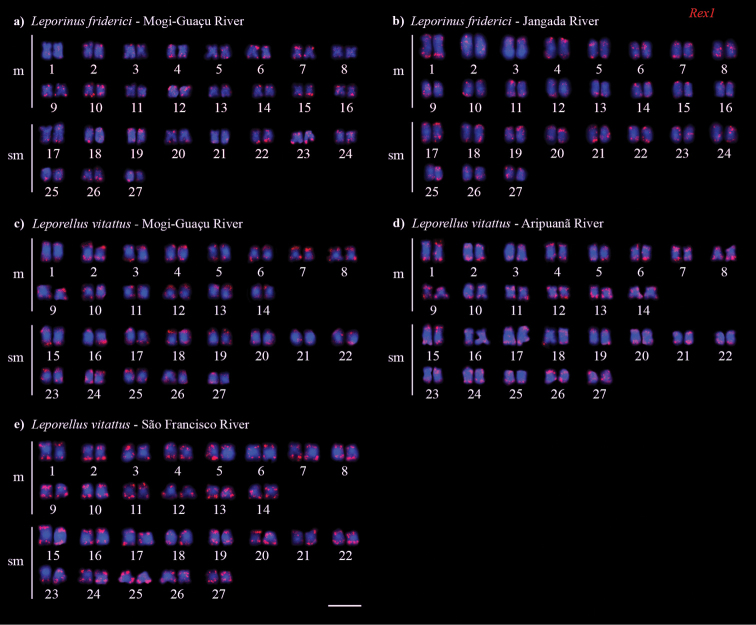
Karyotypes of *Leporinusfriderici* (**a, b**) and *Leporellusvittatus* (**c, d, e**) submitted to fluorescence *in situ* hybridization with *Rex1* probe. Scale bar: 10 µm.

**Figure 5.**
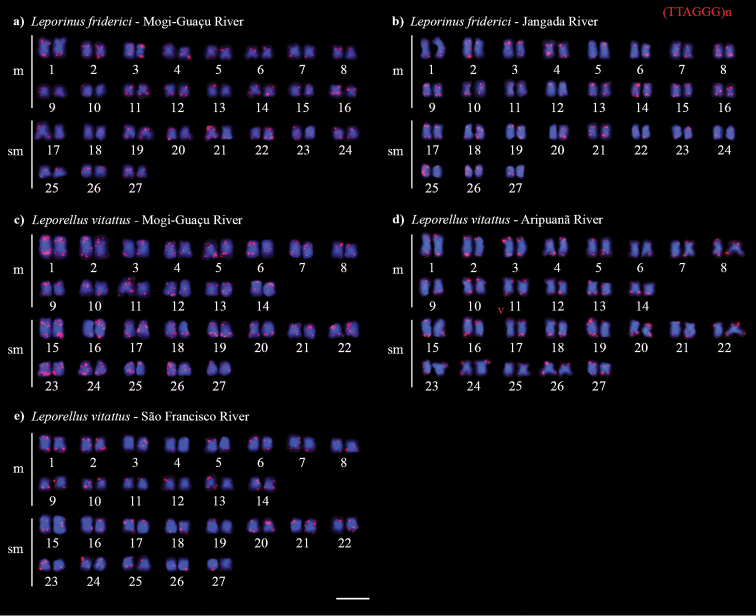
Karyotypes of *Leporinusfriderici* (**a, b**) and *Leporellusvittatus* (**c, d, e**) submitted to fluorescence *in situ* hybridization with (TTAGGG)n probe. Scale bar: 10 µm.

Double-FISH using 5S and 18S rDNA probes detected one 45S rDNA site in the short arm (p) of chromosome pair 1 for both populations of *L.friderici* (Fig. 3a, b). The 5S rDNA was located in the pericentromeric region of chromosome pair 10 and in the short arm (p) of pair 11 for *L.friderici* from the Mogi–Guaçu river (Fig. 3a), whereas it was located in the p arm of chromosome pairs 3 and 11 for *L.friderici* from the Jangada river (Fig. 3b). *Leporellusvittatus* from the Mogi–Guaçu river presented 45S rDNA in the terminal region of the long arm (q) of pair 5, and 5S rDNA was located in the proximal region of 3p pair (Fig. 3c). In *L.vittatus* from the Aripuanã river, the 45S rDNA was located in synteny with 5S rDNA in the chromosome pair 6, with terminal location 6q for 45S rDNA and proximal q arm site for 5S rDNA, and an additional 5S rDNA site in the proximal q arm of pair 8 (Fig. 3d). *Leporellusvittatus* from the São Francisco river presented the 45S rDNA in the terminal region of 6q, and the 5S rDNA in the proximal region of 3p (Fig. 3e). *In situ* mapping of RTE*Rex1* (Fig. 4a–e) and (TTAGGG)n (Fig. 5a–e) showed signals in the terminal regions of all chromosomes for all populations of both *L.friderici* and *L.vittatus*. In *L.vittatus* from the Mogi-Guaçu river, the telomeres signals were tiny in all metaphases analyzed (Fig. 5c).

## Discussion

The present cytogenetic analysis confirmed the conservation of the karyotype macrostructure of 2n = 54 chromosomes in *Leporinusfriderici* and *Leporellusvittatus*, with metacentric and submetacentric chromosomes (FN=108). This karyotype structure is shared by most species belonging to Anostomidae (Galetti Jr et al. 1995, Venere et al. 2004). In addition, *L.friderici* and *L.vittatus* presented small differences in their karyotype formulas resulted of the chromosome rearrangements such as pericentric inversions, translocations or centromere repositioning, which alters the chromosome morphology without any accompanying chromosomal rearrangements (Rocchi et al. 2012).

Some chromosomal markers presented some differentiation within and between species of anostomids. Intraspecific variations were observed in the chromosomal location and quantity of heterochromatin blocks, which were mainly located in pericentromeric regions in *L.vittatus* and terminal positions of chromosomes in *L.friderici*. These heterochromatin distribution in the chromosomes have also been observed for other anostomids (Pereira et al. 2002, Aguilar and Galetti Jr 2008, Barros et al. 2017b). Satellite DNA is one of the components of heterochromatin, which is also enriched in other dispersed repeated elements, including transposons (Mazzuchelli and Martins 2009, Vicari et al. 2010). It is usually accepted that the number of repetitive copies of a heterochromatin block may increase through mechanisms of homologous recombination, TEs invasion, or replication slippage for microsatellite expansion inside heterochromatin (Gray 2000, Kantek et al. 2009, Kelkar et al. 2011, Glugoski et al. 2018). These mechanisms may play a role in the microstructural differentiation of heterochromatin chromosome blocks once no evident large heterochromatic blocks were observed in anostomids species analyzed.

*In situ* location of ribosomal genes showed that these sites were also involved in the chromosomal changes, especially in the studied *L.vittatus* populations. The location of rDNA in different positions and number of chromosomal sites also supports the hypothesis of population differentiation. On the other hand, the location of rDNA sites was observed to be highly conserved in the karyotypes of some anostomids (Martins and Galetti Jr 1999, 2000, 2001). In the present study, consistent differences in the location of rDNA sites were observed between the *L.vittatus* populations evaluated. These differences are exclusive conditions due to population isolation and contribute to genomic diversification in this fish group.

Anostomids usually present only one pair of 45S rDNA (Martins and Galetti Jr 1999), being a common characteristic of this group. Previous studies observed polymorphisms in the number of 45S rDNA sites in *Leporinustaeniatus* Lütken, 1875, *Leporinustrifasciatus* Steindachner, 1876, *Rhytiodusmicrolepis* Kner, 1858 and *Schizodonfasciatus* Spix & Agassiz, 1829 (Barros et al. 2017b). In the present study, although this was also observed, differences in the chromosomal position of 45S rDNA were additionally observed between species, with signals in the terminal region of the p arm for *L.friderici* and in the q arm for *L.vittatus*. The rDNAs usually present high rates of karyotype rearrangements in evolutionary lineages (Symonová et al. 2013). These sequence movements within karyotypes have been proposed to occur by transposition and/or by transposon-mediated by TEs in a non-homologous recombination mechanism (Symonová et al. 2013, Barros et al. 2017a, Glugoski et al. 2018). The *L.vittatus* specimens from the Aripuanã river presented synteny of 45S rDNA and 5S rDNA, in contrast with the specimens from the Mogi–Guaçu and São Francisco rivers and the *L.friderici* populations corroborating to high evolutionary chromosomal change level to rDNA sites. The rDNA synteny was also observed in other anostomids, such as *L.trifasciatus*, *S.fasciatus* and *Laemolytataeniata* (Kner, 1858), showing that it is a recurrent chromosomal characteristic of this group (Barros et al. 2017b).

Recently, some studies have proposed that the dispersal of ribosomal sites and changes in their chromosomal location may affect recombination rates in these specific sites, and that these changes can lead to rapid genome divergence (Symonová et al. 2013). Therefore, these populational chromosome rearrangements due to rDNA transposition could promote differentiation (Symonová et al. 2013, Pucci et al. 2014, Barbosa et al. 2017), which may lead to speciation, as observed in the present study for Anostomidae.

The chromosomal mapping of the non-LTR retrotransposon family *Rex* (*Rex1*, *Rex3* and *Rex6*) has been conducted in the genomes of different teleost species (Volff et al. 1999, 2000, Cioffi et al. 2010, Valente et al. 2011, Borba et al. 2013, Sczepanski et al. 2013, among others). Although they may have a dispersed distribution (Ozouf-Costaz et al. 2004), in most cases, they show strong association with heterochromatic regions (Cioffi et al. 2010, Valente et al. 2011). Overall, the accumulation of RTE sequences in the terminal region of chromosomes has been well documented in *Drosophilamelanogaster* (Meigen, 1830) and in *Sorubimlima* (Bloch & Schneider, 1801), a Neotropical catfish (Eickbush and Furano 2002, Sczepanski et al. 2013). The distribution of *Rex1* sequences in terminal regions of chromosomes in some species of Anostomidae was also detected by *in situ* mapping (Borba et al. 2013). Transpositions and DNA repair by non-homologous recombination involving repetitive sequences in the terminal regions of chromosomes are common during the Rabl configuration of cell division (Schweizer and Loid 1987, Sumner 2003). Furthermore, an efficient strategy to limit the damage caused by retrotransposition in the host genome is to direct the insertion in fairly safe regions, poor in genes, for example in heterochromatin or at telomeres (Okazaki et al. 1995, Zou et al. 1996, Takahashi et al. 1997).

Telomere shortening is usually prevented by telomerase, a reverse transcriptase which adds telomeric repeats to the chromosome ends, thus elongating telomeres (Makarov et al. 1997). The phylogeny involving telomerases and retrotransposons was confirmed after the discovery of a group of retrotransposons, called elements like Penelope, which encodes reverse transcriptase (RT) directly related to an enzyme telomerase (Arkhipova et al. 2003). In *Drosophila*, retrotransposons protect the ends of chromosomes, due to the absence of telomerase, which was possibly lost during evolution (Biessmann et al. 1990). TEs can play a role in the reorganization of the genome being co-opted or exapted to form new genomic functions (Feschotte 2008). This observation suggests the versatility of RT activity in counteracting the chromosome shortening associated with genome replication and that retrotransposons can provide this activity in case of a dysfunctional telomerase. In anostomids analyzed, the (TTAGGG)n sequence was detected in the chromosomal ends, indicating telomerase activity. The short telomere signals detected in *L.vittatus* from the Mogi-Guaçu population can be resulted of the somatic cells telomere shortens with each cell division or, due to inconsistent FISH detection in short telomere sequences. Finally, we observed absence of an interstitial telomeric sequence (ITS), together with the conserved karyotype of 2n = 54 chromosomes, indicating that just non-Robertsonian events may play a role in karyotype diversification in the studied species.

The present study showed intraspecific karyotype variation in populations with isolation of gene flow, and interspecific variation between populations of *L.friderici* and *L.vittatus*. This can be partly explained by genome reorganization due to movement of heterochromatin blocks, ribosomal sites, satellite repetitive sequences, and transposable elements. Our results therefore confirm the conservation of the chromosome macrostructure and indicate karyotypic differentiation at the microstructural level during evolution in Anostomidae.
